# Prevalence and Association of *Mycobacterium avium subspecies paratuberculosis* with Disease Course in Patients with Ulcero-Constrictive Ileocolonic Disease

**DOI:** 10.1371/journal.pone.0152063

**Published:** 2016-03-28

**Authors:** Imteyaz Ahmad Khan, Sucharita Pilli, Surendranath A, Ritika Rampal, Sudhir Kumar Chauhan, Veena Tiwari, Venigalla Pratap Mouli, Saurabh Kedia, Baibaswata Nayak, Prasenjit Das, Govind K. Makharia, Vineet Ahuja

**Affiliations:** 1 Department of Gastroenterology and Human Nutrition, All India Institute of Medical Sciences, New Delhi, India; 2 Department of Pathology, All India Institute of Medical Sciences, New Delhi, India; Universita di Sassari, ITALY

## Abstract

**Background:**

Association of *Mycobacterium avium subspecies paratuberculosis* (MAP) and Crohn’s disease (CD) has been controversial due to contradictory reports. Therefore, we determined the prevalence of MAP in patients with CD and intestinal tuberculosis (ITB) and its association with clinical course.

**Methodology:**

Blood and intestinal biopsies were taken from 69 CD, 32 ITB patients and 41 patients with haemorrhoidal bleed who served as controls. qPCR targeting of MAP-specific IS900 gene was used to detect the presence of MAP DNA. qPCR results were further validated by sequencing. Immunohistochemistry (IHC) was used to detect the presence of MAP antigen in biopsy specimens. CD and ITB patients were followed-up for disease course and response to therapy.

**Principal Findings:**

The frequency of MAP-specific DNA in biopsies by qPCR was significantly higher in CD patients (23.2%, p = 0.03) as compared to controls (7.3%). No significant difference in intestinal MAP presence was observed between ITB patients (12.5%, p = 0.6) and controls (7.3%). MAP presence in blood of CD patients was 10.1% as compared to 4.9% in controls while no patients with ITB were found to be positive (p = 0.1). Using IHC for detection of MAP antigen, the prevalence of MAP in CD was 2.9%, 12.5% in ITB patients and 2.4% in controls. However, long-term follow-up of the patients revealed no significant associations between clinical characteristics and treatment outcomes with MAP positivity.

**Conclusion:**

We report significantly high prevalence of MAP in intestinal biopsies of CD patients. However, the presence of MAP does not affect the disease course and treatment outcomes in either CD or ITB patients.

## Introduction

The aetiology of Crohn’s disease (CD) is unknown and believed to be complex and multifactorial, wherein genetic and environmental factors play a significant role. Several theories have been put forward: (i) a persistent infection, possibly involving *Mycobacteria* (specifically *Mycobacterium avium subspecies paratuberculosis*, MAP), the measles virus, *Listeria spp*., *Escherichia coli* [1,2]; (ii) a defective mucosal barrier[3]; (iii) dysregulation of the host immune response [4]; (iv) genetic susceptibility [5,6]. MAP is a facultative obligate intracellular bacterium that causes Johne’s disease (JD), a chronic progressive granulomatous inflammatory bowel disease, in ruminant animals [7,8]. Several studies have reported contrasting observations regarding the association of MAP and CD in humans [9–14]. India has been an endemic area for intestinal tuberculosis (ITB) in humans as well as JD in ruminants. Traditionally India has been considered as a low prevalence area for CD. However in the last decade there has been a rapid increase in prevalence of inflammatory bowel disease in India and other Asian countries [15]. The phenotypic similarity and coexistence of ITB and CD in Indian population often lead to a diagnostic dilemma in patients with ulcero-constrictive ileocolonic disease [16]. The existence of CD, ITB, and JD in India formed a strong basis to study the prevalence MAP in CD and ITB patients. Therefore, in the present study, we have evaluated the prevalence of MAP in CD and ITB patients using TaqMan based qPCR assays as well as immunohistochemistry (IHC) of tissue biopsies. Subsequently we compared the disease course and treatment outcomes in CD patients with or without MAP infection.

## Materials and Methods

### Study design

The study was approved by Institute ethics committee, All India Institute of Medical Sciences, New Delhi, India (Approval no. IEC/NP-165/2010) and informed written consent was taken from each patient and control subjects. In case of minors/children, written consent was taken from the next of kin, caretakers or guardians. All clinical investigation was conducted according to the principles expressed in the Declaration of Helsinki. One hundred one patients with Ulcero-constrictive disease of the ileocolonic region were recruited from IBD clinic at All India Institute of Medical Sciences, New Delhi, India. The patients included 69 patients with CD and 32 patients with ITB. Forty one patients with suspected haemorrhoidal bleed undergoing sigmoidoscopy served as controls. The diagnosis of CD and ITB were established by standard clinical examinations. The CD patients were treated with corticosteroids, azathioprine, salazopyrine, and 5ASA. The ITB patients were given a trial of ATT (Anti-TB therapy) for more than 6 months. The patients were followed up for clinical course of the disease. Clinical characteristics in CD patients were stratified according to the Montreal Classification and by other features. The respective Montreal Classification status concerning age at diagnosis (<16, 17–40, >40 yrs), behaviour of the disease (Non stricturing, stricturing, penetrating and perianal disease) and location of disease (L1- ileal, L2- colonic, L3- ileocolonic, L4- islolated upper disease) was used. Blood (10 ml) and ileocolonic biopsy samples (25 mg) were taken from the patients.

### DNA extraction

DNA extraction was done from the blood (10 ml) and tissue (25mg) samples by the commercial DNA extraction kit (Qiagen, USA). The amount and purity of DNA were determined by measuring optical density at 260 and 280nm by Picodrop (OD_260/280_ ranged between 1.7–2.0). Extracted DNA was used for target gene amplification by qPCR. Samples were run in duplicate with controls (S1 Fig).

### Quantitative Real-time PCR

A set of primers (IS900 Forward- 5' TAA CGC CCA ACA CAG CAT-3' and IS900 Reverse- 5' CGA AAT CGC TCC TGA ATC AT-3') was designed with Integrated DNA Technologies (IDT) software (Coralville, IA, USA) for detection of IS900 specific sequence. The TaqMan probes (5' TGA TGG CCC TCG ACACC AAA TC 3') was tagged with 6-HEX at the 5^׀^-end as reporter dye and quenched with black hole quencher (BHQ-1) at the 3^׀^ end.

The PCR reaction (20 μl) consisted of 10μl universal 2x probe mix (Thermo Scientific, USA), 2μl of 10x primer and probe mix, 5μl DNA (~ 500 ng) template of interest, and 3μl of water. Amplification and detection were performed on the Stratagene Mx3005p Real time PCR system (Agilent Technologies, USA) with the following profile: 1 cycle of 95°C for 5 min, 35 cycles of 95°C for 10 sec and 60°C for 1 min. Quantitation of the amount of target in unknown samples was measured by C_T_ and using a standard curve prepared with a series of known quantity of the target sequence (S2 Fig).

### IS900-specific semi-quantitative PCR and sequencing

Conventional PCR targeting IS900 gene was carried out using Forward Primer ISO898F (5’ TAA CGC CCA ACA CAG CAT-3’) and Reverse Primer ISO1027R (5’–CGA AAT CGC TCC TGA ATC AT-3’). The PCR reaction mixture (50μl/reaction volume) included 1X PCR buffer, 2.5 mM MgCl_2_, 0.2 mM dNTPs, 0.5 pmol each IS900 forward and reverse primer and 500 ng template DNA. Cycling conditions were 1 cycle of 94°C for 5 min and then 40 cycles of 94°C for 1 min, 58°C for 1 min and 72°C for 1 min followed by 1 cycle of 72°C for 5 min. Amplicons of expected size (124bp) were visualized by 2% agarose gel electrophoresis and ethidium bromide staining and the product was gel purified using QIA-quick gel extraction kit (Qiagen, USA) (S3 Fig).

The gel purified PCR product was sequence confirmed using dye terminator cycle sequencing (DTCS) kit (Beckman-Coulter, USA). The cycle sequencing reaction was carried out in 20 μl reaction volume using 8 μl DTCS mix, 2 μl IS900R (2pmol/ μl), 4 μl template and 6 μl water. The cycle sequencing conditions used was as follows: 30 cycles of 96°C for 20s, 50°C for 20s and 60°C for 4 minutes. The reaction was stopped using 5μl of Stop solution (2μl of 3M sodium acetate, 2μl of 100mM EDTA & 1μl of 20mg/ml) and purified by ethanol precipitation. Sequencing was then performed by capillary gel electrophoresis using Genome Lab GE XP genetic analyzer (Beckman Coulter, USA). The nucleotide sequence was aligned with IS900 reference sequence (X16293) using DNASTAR and BLASTN program.

### IHC for demonstration of MAP antigen in ITB and CD patient biopsy samples

**Fig 1:**
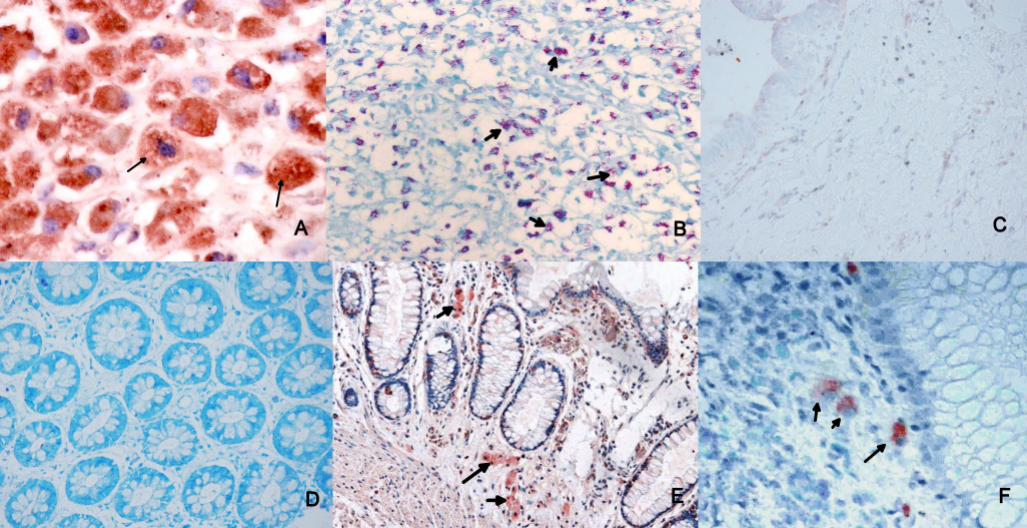
Photomicrograph shows intracytoplasmic granular particulate positivity (arrows) in the macrophages noted in the intestinal control from an experimental animal inoculated with *Mycobacterium paratuberculosis* [Fig A, IHC (MAP) x200]. Ziehl-Neelsen stain shows strongly positive bacilli present in the macrophage cytoplasm in the same section examined [Fig B, AFB stain x100]. Negative control used for MAP IHC stain, does not show any positivity [Fig C, IHC (MAP) x200], the corresponding case is also negative for ZN stain [Fig D, ZN stain x200]. Photomicrographs taken from the cases of Crohn’s and intestinal tuberculosis also show similar positivity in the macrophage cytoplasm (arrows) [Fig E & F, IHC (MAP), Ex100, Fx200].

Four μm thick tissue sections were mounted on glass slides coated with 2% solution of 3-Aminopropyl) triethoxysilane (APTES) solution (Sigma-Aldrich, St. Louis, USA). Immunohistochemistry (IHC) was performed using the indirect immunoperoxidase staining method on paraffin sections. Endogenous peroxidase activity was blocked with 80% methanol and 3% hydrogen peroxide. Non-specific sites were blocked by incubation with normal horse serum (1:5). The tissue sections were then incubated overnight with 1: 50 dilutions of primary antibody (convalescent sheep serum obtained from clinical case of paratuberculosis, Central Sheep and Wool Research Institute, Rajasthan, India).Slides were then incubated with anti-sheep IgG-peroxidase conjugated (dilution 1:100, Sigma, USA) secondary antibody for an hour, followed by washing with phosphate buffer saline solution. The sections were stained with the AEC staining kit (Sigma-Aldrich, St. Louis, USA) and counterstained with haematoxylin stain. Positive control slides containing MAP-positive tissue sections (obtained from intestine of MAP positive sheep) were prepared in similar manner. The slides without incubation with primary antibody was used as appropriate negative control. Slides—mounted with aqueous crystal mount (Sigma-Aldrich, St. Louis, USA) without dehydration procedure—were examined under light microscope for the presence of MAP. MAP was identified as distinct red granules or rods, mostly within macrophage cytoplasm, in the absence of background staining. The representative photomicrogarphs of MAP-negative and -positive samples are shown in Fig 1.

## Results

### Baseline characteristics of CD and ITB patients

Baseline characteristics of the ITB and CD patients along with controls are summarized in Table 1. The CD group consisted of 69 patients (45 males and 24 females), ITB group consisted of 32 patients (20 males and 12 females) and healthy controls consisted of 41 controls (29 males and 12 females). Mean duration of disease was significantly lower in ITB patients as compared to CD patients (p = 0.001). No statistically significant associations were found with respect to the sex, behaviour of disease (non-stricturing, stricturing, penetrating, perianal disease) and location of disease.

**Table 1:** Baseline characteristics of patients with Intestinal Tuberculosis (ITB), Crohn’s disease (CD) and controls.

*Clinical Factor*		*ITB*	*CD*	*Controls*	*P value*
***Number***		32	69	41	
***Sex (M*:*F)***		20:12	45:24	29:12	0.7
***Age at diagnosis***		38.34	37.55	32.02	0.07
***Mean duration of disease (months)***		17.4	63.0		0.001*
***Behavior of disease (Montreal Classification)***	Non stricturing (B1)	13 (40.6%)	41 (59.4%)		0.1
	Stricturing (B2)	19 (59.4%)	27 (39.1%)		
	Penetrating (B3)	0	1 (1.5%)		
	Perianal disease (P)	0	0		
***Location of disease***	Ileal (L1)	9 (28.1%)	22 (31.9%)		0.9
	Colonic (L2)	10 (31.3%)	19 (27.5%)		
	Ileocolonic (L3)	12 (37.5%)	22 (31.9%)		
	Isolated upper digestive (L4)	0	2 (2.9%)		
	L1+L4	1 (3.1%)	1 (1.5%)		
	L2+L4	0	2 (2.9%)		
	L3+L4	0	1 (1.5%)		
***Site of Biopsy***	Rectum	0	1 (1.5%)	2 (4.9%)	0.001*
	Rectosigmoid	1 (3.1%)	1 (1.5%)	2 (4.9%)	
	Sigmoid	0	3 (4.4%)	35 (85.4%)	
	Descending Colon	1 (3.1%)	6 (8.7%)	0	
	Transverse Colon	3 (9.4%)	6 (8.7%)	0	
	Ascending Colon	6 (18.8%)	9 (13.0%)	0	
	Caecum	4 (12.5%)	4 (5.8%)	0	
	Ileocaecal	10 (31.3%)	13 (18.8%)	0	
	Terminal Ileum	7 (21.9%)	26 (37.7%)	2 (4.9%)	
***Granuloma***		5 (15.6%)	3 (4.4%)		0.1

* P<0.05

### High prevalence of MAP-specific DNA in patients with CD

The frequency of MAP-specific IS900 DNA in biopsy samples by qPCR was significantly higher in CD patients (23.2%, p = 0.03) as compared to the controls (7.3%). No significant differences in intestinal MAP presence were observed between ITB patients (12.5%, p = 0.6) and controls (7.3%). MAP-specific IS900 DNA positivity in blood samples of CD patients was 10.1% as compared to 4.9% in controls; while no patients with ITB was found to be positive (p = 0.1). IHC staining for MAP antigen in the intestinal biopsy specimens, revealed the presence of MAP in 2.9% of CD patients, 12.5% of ITB patients and 2.4% of controls. We had also further tested for the presence of other bacteria (*E*.*coli*) to see if the affected region is more susceptible to any pathogen. We checked for presence of *E*.*coli* in mucosal biopsy samples from 32 CD patients and 13 controls. It was seen that *E*.*coli* was detected in 3 of 32 CD patients and none of the controls. Of these 32 CD patients, 16 were MAP positive. Subgroup analysis showed that of these 16 MAP positive CD patients, *E*.*coli* was detected in 2 (12.5%) and of 16 MAP negative CD patients, *E*.*coli* was detected in 1 (6.2%). (S4 Fig). The results of MAP prevalence are summarized in Table 2. Out of 20 MAP positive biopsy samples of patients by real time PCR, 6 were sequenced and similar sequence of IS900 was found which is highly specific for MAP thereby confirming the qPCR results.

**Table 2:** Comparison of MAP positivity between CD, ITB and control subjects.

*Detection method*	*Control (n = 41)*	*CD (n = 69)*	*P value (CD vs control)*	*ITB (n = 32)*	*P value (ITB vs control)*
***DNA Biopsy qPCR***	3 (7.3%)	16 (23.2%)	0.03*	4 (12.5%)	0.6
***DNA Blood qPCR***	2 (4.9%)	7 (10.1%)	0.4	0	0.5
***Immunohistochemistry***	1 (2.4%)	2 (2.9%)	1.0	4 (12.5%)	0.1

* P<0.05

### Disease behaviour and treatment outcomes are independent of presence of MAP in CD and ITB patients

A possible association of MAP-specific IS900 DNA detection with clinical features of CD patients was investigated by stratification of CD cases according to the criteria of Montreal classification system and other characteristics. No significant association was seen with MAP colonization, disease behaviour and treatment outcomes. Table 3 summarizes clinical phenotypes and treatment outcomes in CD patients with and without MAP infection. Table 4 summarizes clinical phenotypes and treatment outcomes in ITB patients with and without MAP infection.

**Table 3:** Comparison of clinical phenotypes and treatment outcomes in CD patients with and without MAP infection.

*Clinical Factor*		*MAP +ve CD*	*MAP–ve CD*	*P value*
***Number***		16	53	
***Sex (M*:*F)***		12:4	33:20	0.3
***Mean duration of disease (months)***		69.2	61.2	0.9
***Mean duration of follow-up (months)***		18.1	16.8	0.9
***Age at Diagnosis (Montreal Classification)***	<16 years (A1)	0	2	1.0
	17–40 years (A2)	10	31	
	>40 years (A3)	6	20	
***Behavior of disease (Montreal Classification)***	Non stricturing (B1)	10	31	1.0
	Stricturing (B2)	6	21	
	Penetrating (B3)	0	1	
	Perianal disease (P)	0	0	
***Location of disease***	Ileal (L1)	5	17	1.0
	Colonic (L2)	5	14	
	Ileocolonic (L3)	6	16	
	Isolated upper digestive (L4)	0	2	
	L1+L4	0	1	
	L2+L4	0	2	
	L3+L4	0	1	
***Site of Biopsy***	Rectum	0	1	0.3
	Rectosigmoid	0	1	
	Sigmoid	0	3	
	Descending Colon	3	3	
	Transverse Colon	2	4	
	Ascending Colon	0	9	
	Caecum	2	2	
	Ileocaecal	3	10	
	Terminal Ileum	6	20	
***Granuloma***		1	2	0.8
***Therapeutic ATT Given***		7	19	0.5
***Oral Steroids***	Yes	8	22	0.9
	No	6	24	
	Not Known	2	7	
***Number of relapses/year***	0	7	28	0.4
	1	5	17	
	2	1	3	
	3	1	0	
	Not Known	2	5	
***Steroid Dependent***	Yes	0	0	0.6
	No	14	48	
	Not Known	2	5	
***Steroid refractory***	Yes	0	1	0.7
	No	14	47	
	Not Known	2	5	
***Intravenous steroid***	Yes	0	2	0.8
	No	14	46	
	Not Known	2	5	
***Azathioprine***	Yes	7	24	1.0
	No	7	24	
	Not Known	2	5	
***Salazopyrine***	Yes	5	14	0.7
	No	9	34	
	Not Known	2	5	
***5 aminosalicyclates***	0	8	27	1.0
	1	6	21	
	Not Known	2	5	

**Table 4:** The association of MAP specific IS900 DNA detection with clinical phenotypes and treatment outcomes of ITB patients.

*Clinical Factor*		*MAP +ve ITB*	*MAP–ve ITB*	*P value*
***Number***		4	28	
***Sex (M*:*F)***		2:2	18:10	0.6
***Mean duration of disease (months)***		15	17.8	0.5
***Mean duration of follow-up (months)***		12	9.9	0.1
***Age at Diagnosis (Montreal Classification)***	<16 years (A1)	0	3	1.0
	17–40 years (A2)	2	13	
	>40 years (A3)	2	12	
***Behavior of disease (Montreal Classification)***	Non stricturing (B1)	1	12	0.6
	Stricturing (B2)	3	16	
	Penetrating (B3)	0	0	
	Perianal disease (P)	0	0	
***Location of disease***	Ileal (L1)	3	6	0.1
	Colonic (L2)	1	9	
	Ileocolonic (L3)	0	12	
	Isolated upper digestive (L4)	0	0	
	L1+L4	0	1	
	L2+L4	0	0	
	L3+L4	0	0	
***Site of Biopsy***	Rectum	0	0	0.7
	Rectosigmoid	0	1	
	Sigmoid	0	0	
	Descending Colon	0	1	
	Transverse Colon	1	2	
	Ascending Colon	0	6	
	Caecum	0	4	
	Ileocaecal	2	8	
	Terminal Ileum	1	6	
***Granuloma***	Yes	0	5	0.5
	No	3	22	
	Not available	1	1	
***Response to ATT (%)***		100%	100%	

## Discussion

India has been an endemic area for ITB in humans and JD in ruminants. Further, there has been a rapid increase in prevalence of inflammatory bowel disease in India and other Asian countries in last decade. Association of MAP, a causal organism of Johne’s disease, with CD has been long debated. Therefore, observing the co-existence of CD, ITB, and JD in India, we took up this study to identify the prevalence of MAP in CD and ITB patients. MAP is a fastidious and slow growing organism and poor recovery of MAP when cultured indicates an extremely long incubation time [7,8,17]. Due to difficulties in growing MAP *in vitro*, molecular methods such as qPCR and sequencing have been developed. This has been greatly aided by the discovery of the IS900 gene sequence which is highly specific for MAP. This IS900 insertion sequence is present in multiple copies of 15–30 per genome. In this study, we used multiple methods to determine the MAP prevalence in CD and ITB patients including TaqMan qPCR from the DNA isolated from blood and intestinal biopsies, along with IHC detection of the MAP antigen in intestinal biopsies. We have demonstrated that CD patients have significant prevalence of MAP in tissue biopsies that is consistent with other observations [9,10,18]. Also, this is the first study to check the association of MAP in ITB patients. However, MAP presence did not show any significant difference between controls and ITB patients. Our findings also suggest that TaqMan based qPCR offers high diagnostic value for detecting MAP as compared to IHC. Further, we identified that tissue specific detection approach is better than using the patient’s blood as the source of MAP DNA. In a previous study [19], the authors investigated a series of freshly obtained mucosal biopsies for presence of MAP, by using mycobacterial growth indicator tube (MGIT) cultures from individuals with CD and controls. They also identified significantly higher MAP DNA level in CD (34 of 37 patients; 92%), than in the controls. However, in the mentioned study, 26% (9/34) of the individuals in the control group were also positive for MAP DNA. The use of freshly processed non-frozen mucosal samples may be one possible reason for the relatively high percentage of positive cases in both groups. In another study conducted by Berstein et al., no association was found between the detection of mucosal MAP DNA by using nested PCR and the presence of CD [11]. Autschbach et al., found that MAP-specific IS900 DNA was present in samples from both diseased small bowel (47%) as well as from the colon (61%) of CD patients [20]. In our study out of 69 CD patients only 2 patients were positive for MAP by IHC. These findings are somewhat consistent with Ellingson et al., where none of the CD patient showed MAP positivity in the paraffin fixed tissues by IHC [21]. In the present study, IHC based determination yielded low and variable MAP detection as compared to MAP-specific PCR. Many other problems of non-specificity or cross-reactivity can be faced while staining the *M*. *paratuberculosis* (MAP) strains by immunohistochemical (IHC) stain. It has to be remembered that, in this study polyclonal serum antibody, raised from naturally MAP infected sheep was used, which shows enhanced analytical sensitivity, as they are mostly not only directed against the cell walled bacillary antigens, but to the other forms of MAP antigens also, as the cell wall deficient spheroplastic and degraded smaller granular antigens. It is known that two physiological forms of MAP exist, i.e., the spherical (spheroplast) or bacillary forms. When MAP infects any host, it often changes in spherical form. The speheroplasts are mostly cell wall deficient (CWD) form of MAP, formed possibly due to the lysosomal activities within a phagolysome; genetic mutations, host bacterial interactions, drug treatments, etc [22]. Fragmented or degraded intracytoplasmic MAP antigens can also be identified with anti-MAP antibody. Reduction of the cross binding of the polyclonal antibody, or background staining was ensured in this study under strict supervision of the pathologist. The identification of all forms of MAP by IHC stain, as bacillary for, spheroplasts or granular antigenic forms (Fig 1A, 1E and 1F); and the ability of the ZN stain to identify only the cell walled bacillary form, are possibly related to the inherent sensitivities of these staining methods. The ZN stain could not stain the spheroplasts, hence, only the bacillary forms were seen (Fig 1B); while the IHC stain, especially using the polyclonal stain, all forms of the bacilli could be stained. ZN stain also might not be able to differentiate between the MAP and other Mycobacterial species, present in human tissues. In this regard, it may be highlighted that, the serum from naturally infected goat with *Mycobacterium paratuberculosis*, was used as the primary antibody in this study, which was confirmed for MAP by culture and sequencing. Hence, it sounds logical to believe that, the antibodies in the serum used, would stain only the *Mycobacterium paratuberculosis* strain. Though was found quite sensitive in our case, the serum antibody used, was not further subjected to species specific specificity tests. Hence, though we believe that the antibody used, definitely has stained the MAP, if at all it also has stained other Mycobacterial species, cannot be definitely commented from this study. Bacillary transformation of the coccoid form has been also described in a host. Antibodies, which are primarily directed against the *Mycobacterial* cell wall lipids, should not have stained the spherical forms. This may also be an explanation of the type of staining seen in this study. As also the PCR technique, identifies the DNA of any length, it performs better, with higher sensitivity and specificity in identifying MAP in tissues. In a study by Delgado F, et al, the authors had detected that IHC technique had better sensitivity in identifying the MAP, than the direct *in-situ* PCR method they used [23]. There are studies, where using the monoclonal antibodies against the specific MAP antigens, the authors could not demonstrate any diagnostic advantage over the polyclonal stains [22]. However, the outcome of the IHC staining depends on various factors, as the fixative used, time of fixation, staining protocols, etc., hence variability of the staining can be an inherent problem of this technique.

Another goal of the present study was to identify the predictive value of MAP colonization with clinical features and disease course in CD patients. The careful classification of CD patients based on MAP-DNA positivity showed no significant association of clinical disease course and treatment outcomes with MAP status. Similar to our study Autschbach et al., also found no firm association between MAP specific IS900 detection rates and clinical phenotypic characteristics in CD [20]. As CD is governed by multiple factors and it aetiology is complex, disease course may be influenced by several other factors including genetic predisposition and other environmental determinants. Taken together, the high prevalence of MAP DNA in intestinal biopsies of CD patients indicates a possible association of MAP with CD but it remains unclear whether this infection is causal or secondary to underlying immune deficiencies in CD patients.The main strength of this study are the use of qPCR based MAP detection followed by sequencing and a long-term follow-up of MAP positive and MAP negative patients. However, a study with more number of patients will be required for conclusive results and to better interpret the role of MAP in disease course of CD.

## Supporting Information

S1 Fig Agarose gel Electrophoresis- Isolated genomic DNA was detected on 0.8% agarose gel. (DOCX)

S2 Fig Standard curve and amplification plot for qPCR TaqMan assay of MAP IS900 gene quantification. (a) Standard curve generated by plotting the known DNA concentrations of standard DNA(IVRI strain) template (3x10^10^–3x 10^2^ copies) against the corresponding threshold cycles (Ct values) with MxPro^TM^ QPCR software. (b) Representative amplification plots for qPCR assay of MAP IS900 gene quantification for blood and biopsy samples of patients and controls.
(DOCX)

S3 Fig Conventional PCR gel showing IS900 DNA product. Lane 0 consisted of 100bp DNA ladder while the lane marked 1 to 4 was IS900 DNA product, which was 124bp in length. Lane 5 was NTC (non template control).
(DOCX)

S4 Fig E.coli co-infection in MAP +ve and MAP–ve CD patients. (a) Representative gel picture showing E.coli DNA in MAP positive and MAP negative CD patients. (b) Prevalence of E.coli in MAP positive and MAP negative CD patients.
(DOCX)
