# Supplementary material for: Prevalence and Association of Mycobacterium avium subspecies paratuberculosis with Disease Course in Patients with Ulcero-Constrictive Ileocolonic Disease
Source: PLoS One. 2016 Mar 28;11(3):e0152063. doi: 10.1371/journal.pone.0152063 (PMC4809507; doi:10.1371/journal.pone.0152063)
Supplement: S1 Fig — (DOCX) [file pone.0152063.s001.docx]

**S1 Fig**. : **Agarose gel Electrophoresis**- Isolated genomic DNA was detected on 0.8% agarose gel


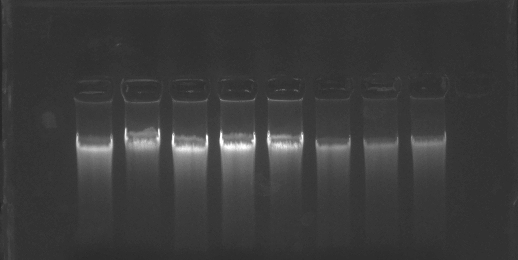


Genomic DNA bands
