# Supplementary material for: Prevalence and Association of Mycobacterium avium subspecies paratuberculosis with Disease Course in Patients with Ulcero-Constrictive Ileocolonic Disease
Source: PLoS One. 2016 Mar 28;11(3):e0152063. doi: 10.1371/journal.pone.0152063 (PMC4809507; doi:10.1371/journal.pone.0152063)

**S2 Fig. : Standard curve and amplification plot for qPCR TaqMan assay of MAP IS900 gene quantification**

1. Standard curve generated by plotting the known DNA concentrations of standard DNA(IVRI strain) template (3x10^10^ – 3x 10^2^ copies) against the corresponding threshold cycles (Ct values) with MxPro^TM^ QPCR software


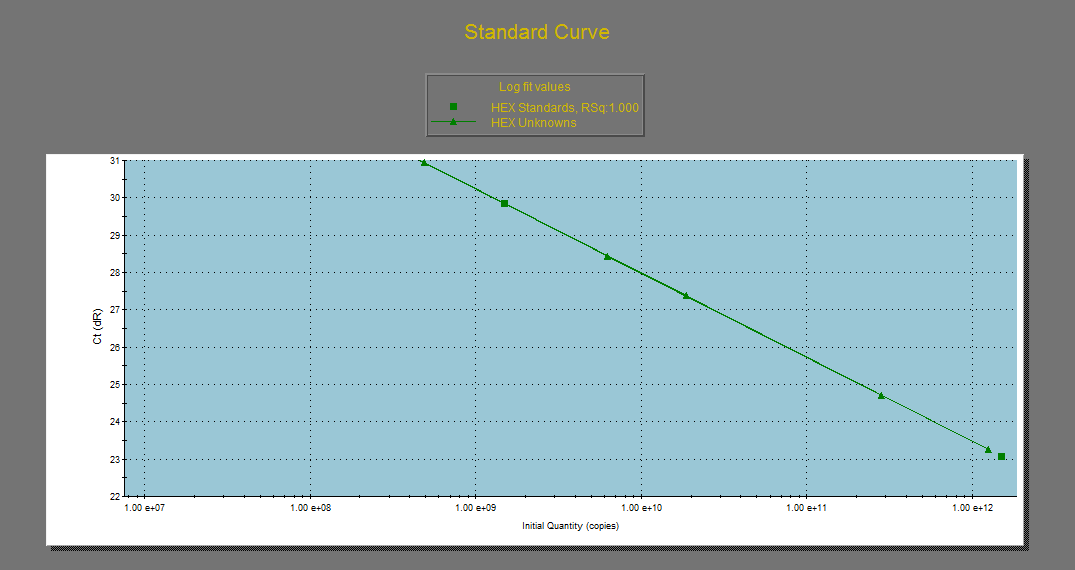


1. Representative amplification plots for qPCR assay of MAP IS900 gene quantification for blood and biopsy samples of patients and controls.


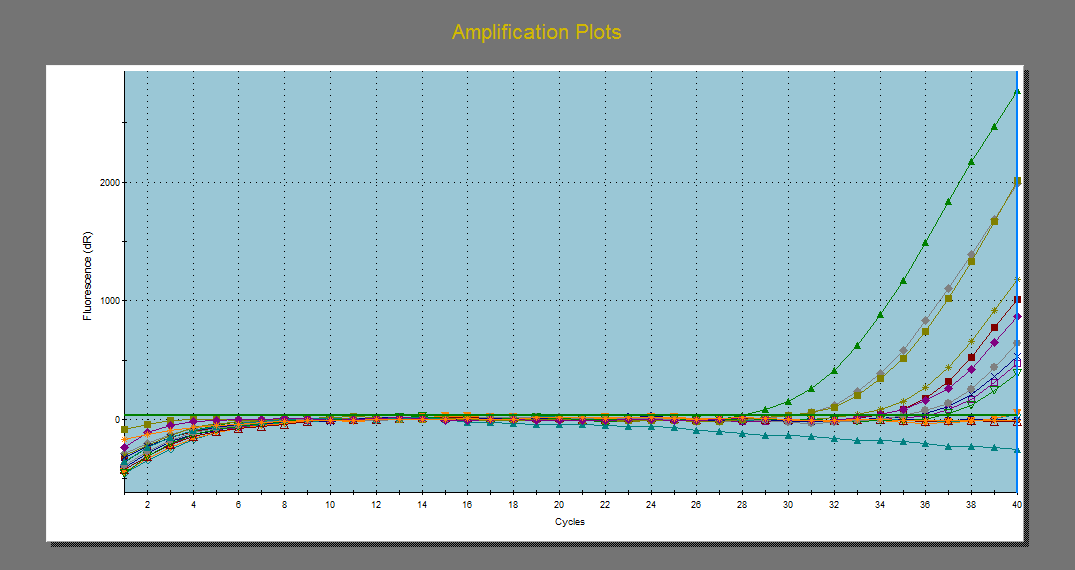

Supplement: S2 Fig — (a) Standard curve generated by plotting the known DNA concentrations of standard DNA(IVRI strain) template (3x10^10^–3x 10^2^ copies) against the corresponding threshold cycles (Ct values) with MxProTM QPCR software. (b) Representative amplification plots for qPCR assay of MAP IS900 gene quantification for blood and biopsy samples of patients and controls. (DOCX) [file pone.0152063.s002.docx]
