# Supplementary material for: Prevalence and Association of Mycobacterium avium subspecies paratuberculosis with Disease Course in Patients with Ulcero-Constrictive Ileocolonic Disease
Source: PLoS One. 2016 Mar 28;11(3):e0152063. doi: 10.1371/journal.pone.0152063 (PMC4809507; doi:10.1371/journal.pone.0152063)
Supplement: S3 Fig — Lane 0 consisted of 100bp DNA ladder while the lane marked 1 to 4 was IS900 DNA product, which was 124bp in length. Lane 5 was NTC (non template control). (DOCX) [file pone.0152063.s003.docx]

**S3 Fig.** : **Conventional PCR gel showing IS900 DNA product**. Lane 0 consisted of 100bp DNA ladder while the lane marked 1 to 4 was IS900 DNA product, which was 124bp in length.Lane 5 was NTC (non template control)

**
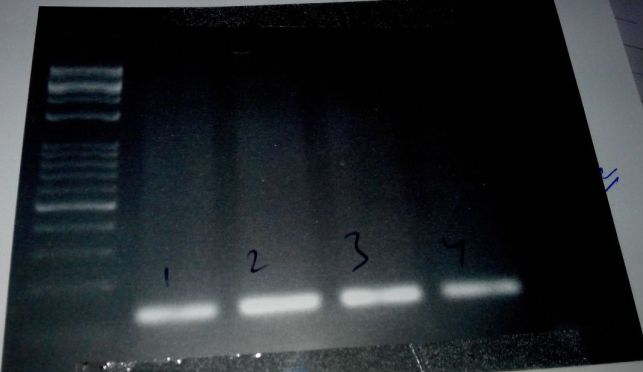
**

NTC

124bp IS900 DNA

DNA ladder
