# Supplementary material for: Prevalence and Association of Mycobacterium avium subspecies paratuberculosis with Disease Course in Patients with Ulcero-Constrictive Ileocolonic Disease
Source: PLoS One. 2016 Mar 28;11(3):e0152063. doi: 10.1371/journal.pone.0152063 (PMC4809507; doi:10.1371/journal.pone.0152063)
Supplement: S4 Fig — (a) Representative gel picture showing E.coli DNA in MAP positive and MAP negative CD patients. (b) Prevalence of E.coli in MAP positive and MAP negative CD patients. (DOCX) [file pone.0152063.s004.docx]

**S4 Fig.** : **E.coli co-infection in MAP +ve and MAP –ve CD patients.**

1. Representative gel picture showing E.coli DNA in MAP positive and MAP negative CD patients.


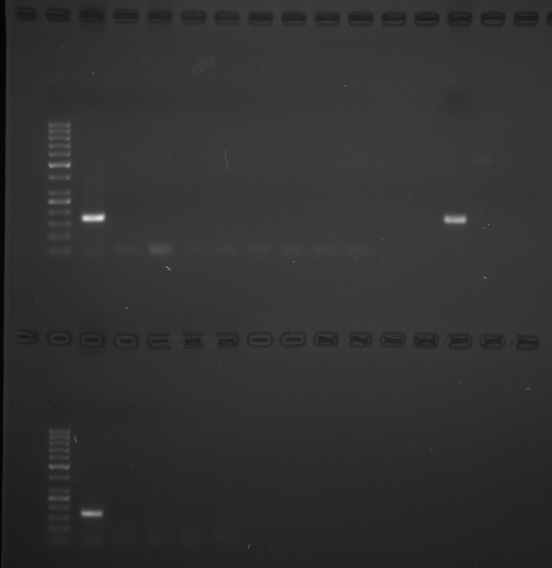

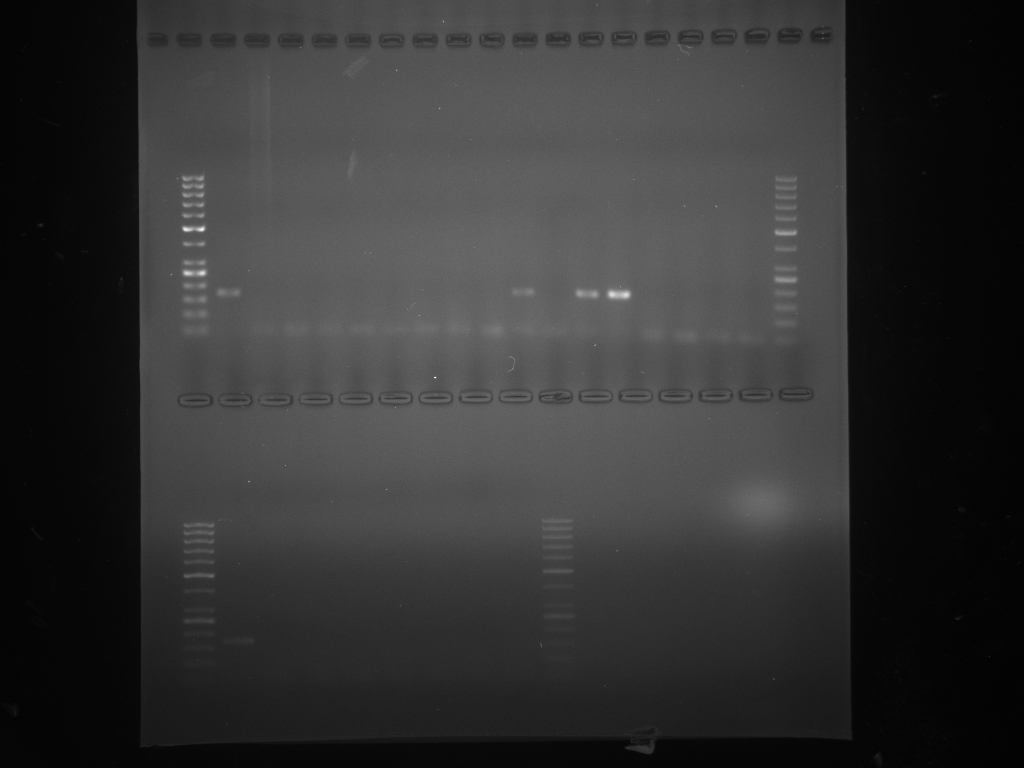


**+ve control**

**+ve control**

**50bp ladder**

**50bp ladder**

**MAP +ve sample**

**MAP –ve sample**

(b) Prevalence of E.coli in MAP positive and MAP negative CD patients

| **E.coli Detection** | **MAP +ve patients** | **MAP –ve patients** |
| --- | --- | --- |
| **Crohn’s Disease** | **2/16 (12.5%)** | **1/16 (6.2%)** |
| **Controls** | **0/3** | **0/10** |
